# Crosstalk between glutathione and melatonin in chromium detoxification in sweet potato revealed by high-throughput sequencing and physio-biochemical profiling

**DOI:** 10.3389/fpls.2026.1767742

**Published:** 2026-03-02

**Authors:** Sunjeet Kumar, Muhammad Ikram, Zhi Huang, Yi Liu, Yongping Li, Mengzhao Wang, Guopeng Zhu

**Affiliations:** 1 School of Breeding and Multiplication (Sanya Institute of Breeding and Multiplication), Hainan University, Sanya, Hainan, China; 2 Key Laboratory for Quality Regulation of Tropical Horticultural Crops of Hainan Province, School of Tropical Agriculture and Forestry, Hainan University, Haikou, China

**Keywords:** chromium stress, detoxification, glutathione, hub genes, photosynthesis, sweet potato, transcriptome analysis

## Abstract

Chromium (Cr) contamination severely inhibits plant productivity, primarily by disrupting photosynthetic performance, growth, and inducing oxidative stress. This study investigated the comparative molecular mechanisms by which exogenous glutathione (GSH) and melatonin (MT) confer tolerance to Cr stress in sweet potato using an integrated transcriptomic approach. Transcriptome analysis identified 7,734 differentially expressed genes (DEGs) across Cr stress and mitigator treatments. The GO and KEGG pathway enrichment analyses showed that DEGs were mainly enriched in GO terms, such as photosynthesis, carbon fixation, and cell-wall organization, as well as pathways including MAPK signaling, glutathione metabolism, plant hormone signal transduction, and membrane/vesicle transport. These DEGs were subjected to cluster analysis, and four major expression clusters (C1-C4) were identified, with DEGs ranging from 1,234 to 3,285. GSH-specific protection was associated with Cluster C4, enhancing genes involved in ROS defense, H_2_O_2_ response, pyruvate metabolism, and ER protein processing, indicating improved redox and metabolic control Network analysis identified 30 potential key hub genes, including the growth regulator *GA20OX1*, the chlorophyll synthesis enzyme *HEMA1*, and the vacuolar transport aquaporin *TIP2-1*. These results suggest that GSH induces a stronger transcriptional response by effectively mitigating Cr-induced damage and strengthening a redox-centered defensive metabolic network. This identifies actionable molecular targets for crop improvement through breeding and genetic engineering.

## 1 Introduction

Hexavalent chromium (Cr) is a highly mobile, persistent, and widespread environmental pollutant released primarily from industrial processes, such as electroplating and leather tanning (Georgaki et al., 2023; Zulfiqar et al., 2023; Suyanto et al., 2025). Cr(VI) is significantly more toxic than [Cr(III)], acting as a strong oxidant, a proven human carcinogen, and a potent mutagen due to its ability to disrupt cellular redox balance and induce DNA damage in living organisms (Dhal et al., 2013). The presence of Cr(VI) in agricultural soils and water severely impairs microbial communities, affects soil fertility, and threatens ecosystem function (Zheng and Duan, 2022; Georgaki et al., 2023). Because plants are the primary interface between contaminated soils and the human food chain, reducing Cr(VI) uptake and toxicity in plants is critical for protecting both crop productivity and food safety. However, conventional physical and chemical remediation strategies, such as precipitation, ion exchange, and membrane separation, are often costly, generate secondary waste, or fail to address the dynamic redox transformations of Cr in the environment (Beig et al., 2023; Yadav et al., 2023). Therefore, it is necessary to develop effective, low-cost, and sustainable biological mitigation approaches, particularly those utilizing plants and representative bioactive molecules—such as antioxidants and signaling/metal-binding compounds (e.g., glutathione (GSH) and melatonin (MT))—to limit Cr bioavailability and toxicity.

Sweet potato (*Ipomoea batatas* L.) is a vital global food crop, recognized as a strategic staple in Asia and Africa, valued for its exceptional yield, resilience, and rich nutritional profile, including high content of β-carotene, vitamins, minerals, and dietary fiber (Heider et al., 2021; Kumar et al., 2022b; Ahmed et al., 2024). The tubers and leaves of sweet potato are widely consumed, and the accumulation of toxic heavy metals (HMs) directly compromises both food and nutritional security (Kumar et al., 2022b, 2024). As a root/tuber crop, sweet potato is more likely a acquire metals directly from soil, underscoring its relevance for assessing Cr accumulation risk and developing mitigation strategies. Exposure to Cr stress has been shown to inhibit sweet potato growth severely, compromise photosynthetic efficiency, disrupt root development, and promote cellular oxidative damage through excessive reactive oxygen species (ROS) production (Kumar et al., 2022a; Sapakhova et al., 2023). Therefore, understanding the intrinsic and inducible tolerance mechanisms in sweet potato is essential for developing effective strategies to maintain yields and ensure safe production in Cr-contaminated environments.

Among plant-based mitigation strategies, the exogenous application of small bioactive molecules has emerged as a promising method to enhance HM tolerance (Singhal et al., 2023; Charagh et al., 2024; Zhang et al., 2024). Melatonin (MT), a pleiotropic molecule, improves tolerance by enhancing overall antioxidant systems, modulating signaling pathways, and influencing metal uptake and compartmentation (Nawaz et al., 2018; Sun et al., 2021; Altaf et al., 2023; Sharma et al., 2024). In contrast, reduced glutathione (GSH), a central low-molecular-weight thiol, plays a more direct role in metal detoxification (Yadav, 2010; Hossain et al., 2012; Ali et al., 2020). GSH can directly scavenge ROS, sustain the ascorbate-glutathione (AsA-GSH) cycle, and serve as the direct precursor for phytochelatins (PCs), which chelate and sequester metals into the vacuole (Hossain et al., 2012). Our previous physiological and biochemical study depicted that both MT and GSH significantly improved sweet potato growth and reduced oxidative damage under Cr stress. Critically, this study concluded that exogenous GSH provided a superior protection to MT, reducing Cr accumulation and oxidative injury more effectively (Kumar et al., 2024). However, the specific transcriptional basis for this differential effectiveness between MT and GSH in sweet potato remains largely unknown.

At the molecular level, plant survival under HM stress relies on the coordinated action of a complex regulatory network. This response involves three primary lines of defense: first, chelation and sequestration via the synthesis of thiols such as GSH, PCs, and metallothioneins (MTs) (Joshi et al., 2015; Liu et al., 2019; Raychaudhuri et al., 2021). Secondly, transport mechanisms, mediated by transporter families, such as HMA, NRAMP, ZIP, and ABC transporters, which control metal uptake, long-distance translocation, and vacuolar compartmentalization (Moravčíková, 2023; Lin et al., 2024). Thirdly, antioxidant defense through the induction of enzymatic and non-enzymatic systems to neutralize ROS. These adaptive changes are tightly controlled by intricate signaling pathways, including MAPK cascades and Ca^2+^ signaling, which activate a group of transcription factors (TFs) (Chen et al., 2017). Key TF families, notably MYB, WRKY, NAC, and bZIP, serve as central regulatory hubs, orchestrating the expression of downstream genes involved in detoxification, redox homeostasis, and secondary metabolism to confer enhanced tolerance (Singh et al., 2016; Xu et al., 2017; He et al., 2020; Zhang et al., 2025).

Based on previous evidence that GSH is the superior mitigator in sweet potato under Cr stress, this study employed RNA-seq combined with comprehensive bioinformatics analysis to dissect the underlying molecular mechanisms. We utilized differential expression analysis (DEA), functional enrichment (GO and KEGG), and Weighted Gene Co-expression Network Analysis (WGCNA) to compare the transcriptional profiles of sweet potato under Cr stress and mitigator treatments. The primary objectives were to (i) characterize the global transcriptional reprogramming induced by each treatment; (ii) identify biological functions and metabolic pathways, and co-expression modules significantly associated with key physiological parameters and Cr accumulation; and (iii) mine GSH-associated hub genes and TFs that underpin the enhanced tolerance. We hypothesized that exogenous GSH would act as a more potent and more distinct transcriptional modulator than MT, specifically activating detoxification, redox, and metal transport pathways that contribute to the superior mitigation of Cr toxicity in sweet potato.

## 2 Materials and methods

### 2.1 Plant material and growth conditions

Sweet potato (*I. batatas* L.) cv. ‘Haida HD7791’ was used in this study, following the hydroponic protocol described by (Kumar et al., 2024). Briefly, stem cuttings were surface-sterilized with Carbendazim (1 g L^-1^), rooted in distilled water, and then transferred to half-strength Hoagland nutrient solution. The nutrient solution pH was monitored daily with a calibrated pH meter and adjusted to 6.0 ± 0.5 with dilute NaOH or HCl in a controlled growth room (25 to 27°C, 16 h light/8 h dark). After 6 days, seedlings were subjected to pre-treatments with MT (1 µM) and GSH (2 mM) for 7 days, followed by exposure to 40 μM Cr(VI) applied as K_2_Cr_2_O_7_ in the same nutrient solution. The RNA-seq experiment used four treatments: CK, Cr, MT + Cr (MC), and GSH + Cr (GC), each with three biological replicates of six plants per replicate. Growth, physiological and biochemical traits, including plant height, leaf number and area, shoot and root fresh/dry weight, relative water content (RWC), root architectural traits, gas-exchange parameters, chlorophyll fluorescence and pigments, ROS and MDA, electrolyte leakage, osmoprotectants (soluble sugars and proline), antioxidant metabolites (GSH, GSSG, AsA, phenolics, and flavonoids), antioxidant enzymes, stomatal traits, and Cr uptake/translocation, were measured as previously described by (Kumar et al., 2024), which were subsequently used as external traits in the WGCNA analysis.

### 2.2 RNA isolation, library preparation, and sequencing

For RNA-seq, leaf tissue was collected from plants grown under the four treatments (CK, Cr, MC, and GC) at the same developmental stage used for physiological measurements. For each treatment, samples from multiple plants within a replicate pot were pooled to form one biological replicate, yielding 12 libraries in total. Total RNA was extracted using a plant RNA isolation kit (RNA Easy Fast Plant Tissue, Tiangen Biotech, Beijing, China) according to the manufacturer’s instructions. RNA integrity and purity were evaluated by 1% agarose gel electrophoresis, spectrophotometry (A260/280 and A260/230 ratios), and an Agilent 2100 Bioanalyzer. For each qualified sample, mRNA was purified from 1-2 µg of total RNA using oligo(dT) magnetic beads, fragmented, and reverse transcribed to first-strand cDNA using random hexamer primers, followed by second-strand synthesis. After end repair, A-tailing, adapter ligation, and PCR enrichment, size-selected cDNA fragments were used to construct strand-specific libraries following the standard Illumina protocol. The libraries were quantified and sequenced on an Illumina HiSeq X Ten platform (Illumina, San Diego, CA, USA) to generate paired-end reads.

### 2.3 Quality control and preprocessing of reads

FastQC v0.11.9 (https://www.bioinformatics.babraham.ac.uk/projects/fastqc/) was used to assess raw read quality, including per-base quality scores, GC content, sequence length distribution, and adapter contamination. Raw reads were then filtered with Trimmomatic v0.39 (Bolger et al., 2014) to remove Illumina adapter sequences, trim low-quality bases, discard reads containing > 10% unknown bases (N), and eliminate reads shorter than 36 bp. Reads were reevaluated via FastQC to confirm the removal of low-quality and contaminated sequences, and only high-quality clean reads were retained for downstream analyses. Clean reads from each library were aligned to the sweet potato reference genome Xushu 18 genome with the annotation v1.0.a2 using HISAT2 (v2.2.1) with default parameters (Kim et al., 2019), and the resulting SAM files were converted, sorted, and indexed as BAM files with SAMtools v1.10 (Danecek et al., 2021). Gene-level read counts were obtained with featureCounts v2.0.0 (Liao et al., 2014) using the corresponding genome annotation, while transcript-level abundance was estimated as transcripts per million (TPM) with StringTie v2.2.1 (Pertea et al., 2015) to provide normalized expression values for subsequent differential expression and co-expression analyses. RNA-seq data has been submitted to the National Genomics Data Center (NGDC) database (accession number: PRJCA053307; https://ngdc.cncb.ac.cn).

### 2.4 Differential gene expression and cluster analyses

Genes with very low mean normalized expression were removed, and 68,922 genes were retained for downstream analysis. Principal component analysis (PCA) and hierarchical clustering were performed in R 4.4.1 (http://www.r-project.org/) to assess sample variability and detect potential outliers. Differential expression analysis for all pairwise contrasts: T1 (Cr vs Ck), T2 (GC vs Ck), T3 (MC vs Ck), T4 (GC vs Cr), T5 (MC vs Cr), and T6 (GC vs MC), was carried out with DESeq2 (Love et al., 2014) based on raw gene-level counts. P-values were adjusted using the Benjamini–Hochberg method, and genes with |log2FC| ≥ 1 and FDR ≤ 0.05 were defined as differentially expressed. Heatmaps and volcano plots were generated using the R packages pheatmap and ggplot2, respectively. For expression clustering, all DEGs were clustered using visCluster in the ClusterGVis package v0.1.0 (https://github.com/junjunlab/ClusterGVis), with normalized TPM values as input, to identify major expression patterns across CK, Cr, GC, and MC treatments. Transcription factors among DEGs and hub genes were annotated using PlantTFDB (Jin et al., 2017).

### 2.5 Functional annotation and enrichment analysis

All expressed genes and DEGs were functionally annotated against public databases, including NCBI non-redundant (NR), Swiss-Prot, Gene Ontology (GO), and Kyoto Encyclopedia of Genes and Genomes (KEGG), using BLAST-based workflows and eggNOG-mapper. Functional enrichment analysis of DEGs, co-expression clusters, and trait-related WGCNA modules was performed in R 4.4.1 with clusterProfiler (Wu et al., 2021), considering GO biological process (BP), molecular function (MF), cellular component (CC) terms, and KEGG pathways (Kanehisa and Goto, 2000). The full set of expressed genes served as the background, and the Benjamini–Hochberg correction was applied to control the FDR; GO terms/pathways with adjusted p (FDR) ≤ 0.05 and at least 2 genes per term were considered significantly enriched. The top enriched categories were visualized using ggplot2 and enrichplot.

### 2.6 Weighted gene co-expression networks analysis

Weighted gene co-expression network analysis (WGCNA) was performed in R 4.4.1 using the WGCNA package v1.73 (Langfelder and Horvath, 2008) to identify modules associated with Cr tolerance and glutathione-mediated physio-chemical improvements. Genes with extremely low expression or variance were removed, and the remaining expression matrix (selected top 25%) across all samples was used as input. A signed network was constructed by calculating pairwise Pearson correlation coefficients between genes and transforming them into an adjacency matrix using a soft-thresholding power selected with the pickSoftThreshold function to approximate scale-free topology (R^2^ close to 0.8). The adjacency matrix was then converted into a topological overlap matrix (TOM), and the corresponding dissimilarity (1–TOM) was used for average-linkage hierarchical clustering. Gene modules were defined using the dynamic tree cut algorithm with a minimum module size of 30 genes, and closely related modules (eigengene dissimilarity < 0.25) were merged. Module eigengenes (MEs) were correlated with the panel of growth, physiological, and biochemical traits. Modules significantly associated with improved photosynthetic and antioxidant traits and/or reduced oxidative damage and Cr load were considered GC-related modules. Within these modules, key genes were defined based on high module membership (MM) and gene significance (GS) values (e.g., |MM| ≥ 0.8 and |GS| ≥ 0.3). The significant modules were exported to Cytoscape (Otasek et al., 2019), and the CytoHubba plugin (Chin et al., 2014) was used to calculate node degree and identify the top 30 hub genes, which were further analyzed as candidate regulators underlying glutathione-driven physio-chemical improvements under Cr stress.

### 2.7 Validation of genes via RT-qPCR

To validate the RNA-seq results, eight potential candidate genes were selected for RT-qPCR analysis. Leaf samples from the four treatments mentioned above were collected in parallel with those used for RNA-seq, with three biological replicates per treatment. Total RNA was extracted as described above, and 1 µg of DNase-treated RNA was reverse transcribed using a commercially available cDNA synthesis kit (Fastking gDNA Dispelling RT SuperMix, Tiangen Biotech, Beijing, China) according to the manufacturer’s instructions. Gene-specific primers were designed in Primer3 software, and a housekeeping gene (*Actin*) was used as an internal reference. qRT-PCR was performed using a MA-6000 PCR system (Suzhou Yarui Biotechnology, China). Each sample was run in triplicate. Relative expression levels were calculated using the 2^-ΔΔCt^ method. The primers of selected genes are listed in **Supplementary Table 1**.

## 3 Results

### 3.1 Overview of transcriptomic data and hypothesis development

**Figure 1:**
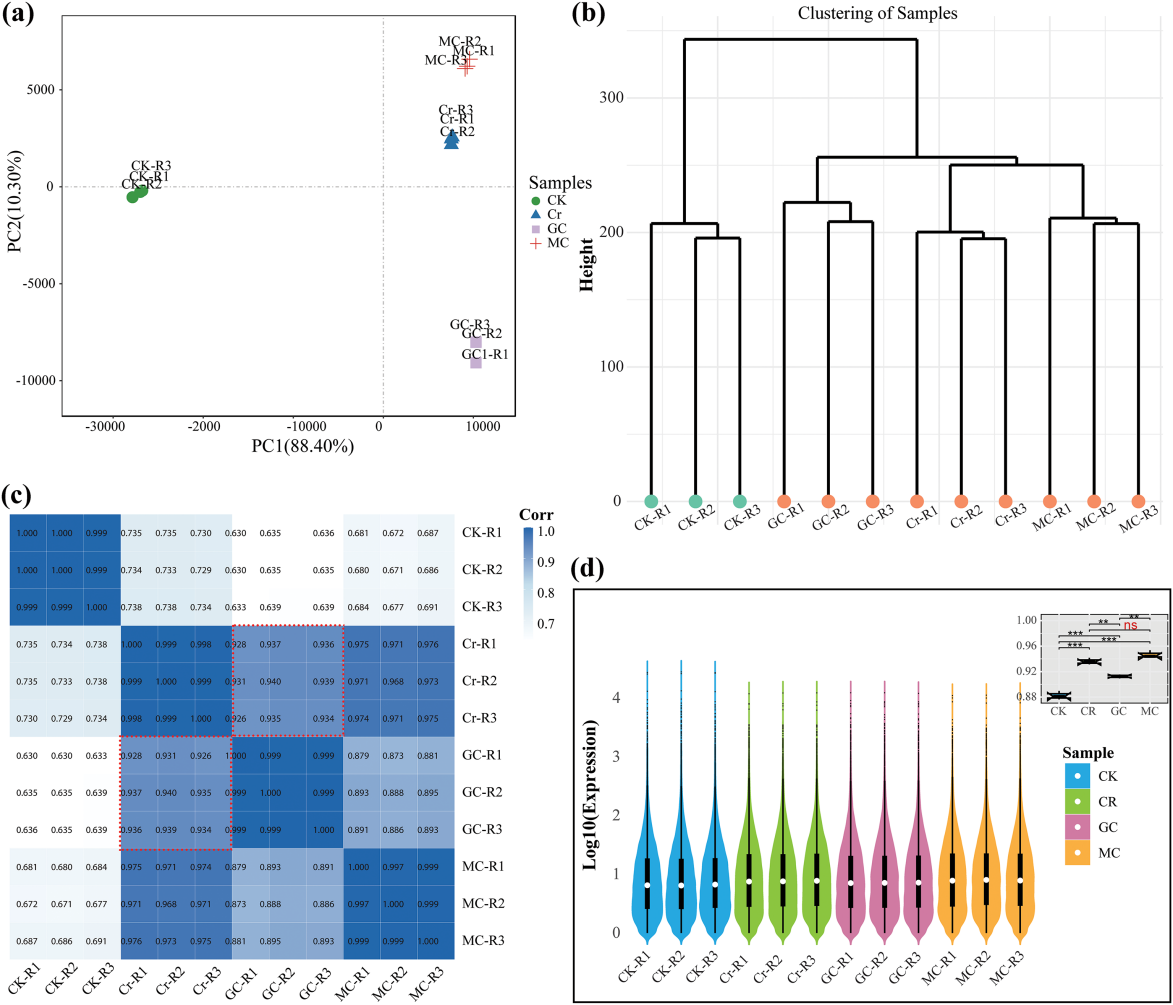
Overview of the global transcriptome relationships and sample clustering among the four treatment groups. The figure assesses the degree of similarity and separation between the transcriptomic profiles of the control (CK), chromium stress (Cr), glutathione-mitigated Cr stress (GC), and melatonin-mitigated Cr stress (MC) groups. **(a)** Principal component analysis (PCA) of transcriptome profiles. **(b)** Hierarchical clustering of samples based on distances, which illustrates the transcriptional proximity of the groups, showing that GC forms a distinct cluster separate from the Cr/MC branch. **(c)** Pearson correlation matrix. **(d)** Boxplots of TPM distributions, which indicate the significant differences in gene expression variability and magnitude among all pairs.

We sequenced 12 RNA-Seq libraries to decode the molecular crosstalk between GSH and MT to mitigate the effect of Cr stress. Across 12 libraries, sequencing quality was high, with 99.60% to 99.70% of raw reads retained as clean reads (**Supplementary Table 2**). The overall genome alignment was consistent across treatments, with total mapped reads ranging from ~84% to ~86%, of which about 64% to 66% were uniquely mapped and ~20% mapped to multiple loci. Unmapped reads remained low (14% to 16%), indicating reliable RNA-seq data suitable for downstream differential expression analysis. Given the high genome complexity of sweet potato (a highly heterozygous hexaploid, 2n = 6x = 90), mapping rates of 84–86% are expected, as polyploidy and repetitive/homologous sequences can reduce unique alignment. PCA accounted for 88.4% and 10.3% of the total variance along PC1 and PC2, respectively (**Figure 1a**). The CK clustered together, confirming replicate consistency. Cr stress induced a substantial transcriptional shift relative to CK, while MC clustered near Cr, indicating limited transcriptomic deviation from Cr stress. In contrast, GC samples were clearly separated from Cr along PC1, demonstrating a distinct expression pattern. These results suggest that GSH exerts a more pronounced effect on transcriptomic reprogramming during Cr detoxification than melatonin. Hierarchical clustering of transcriptomes revealed a clear treatment-level structure (**Figure 1b**), with biological replicates clustering tightly within each condition, confirming reproducibility. GC formed a distinct branch, indicating a transcriptional program that diverges from both CK and Cr stress. In contrast, MC grouped with Cr on the same major branch, consistent with a MT response that remains close to the Cr-stress state. Pairwise Pearson correlations supported this hypothesis (**Figure 1c**). MC vs Cr showed a robust correlation (r = 0.97), indicating minimal deviation from chromium-induced expression. GC vs Cr correlations were lower (r = 0.92–0.94), indicating a larger GC-driven reprogramming of the Cr response. GC vs MC correlations were lower still (r = 0.87–0.89), highlighting distinct effects of GSH and MT under identical Cr exposure. Compared with CK, GC showed a lower positive correlation than either Cr or MC, consistent with GC entering a unique detoxification state rather than reverting to a control-like profile. The boxplots of all groups showed variation among the samples and treatment groups (**Figure 1d**). Mean expression comparisons were significant for all pairwise contrasts (p ≤ 0.05) except MC vs Cr, which was non-significant, again indicating that MT-treated leaves were close to Cr stress, whereas GSH drives a more distinct adjustment. These results demonstrated that GC has a stronger, more distinct expression profile than Cr and MC, partially supporting our hypothesis.

### 3.2 Identification of differentially expressed genes

**Figure 2:**
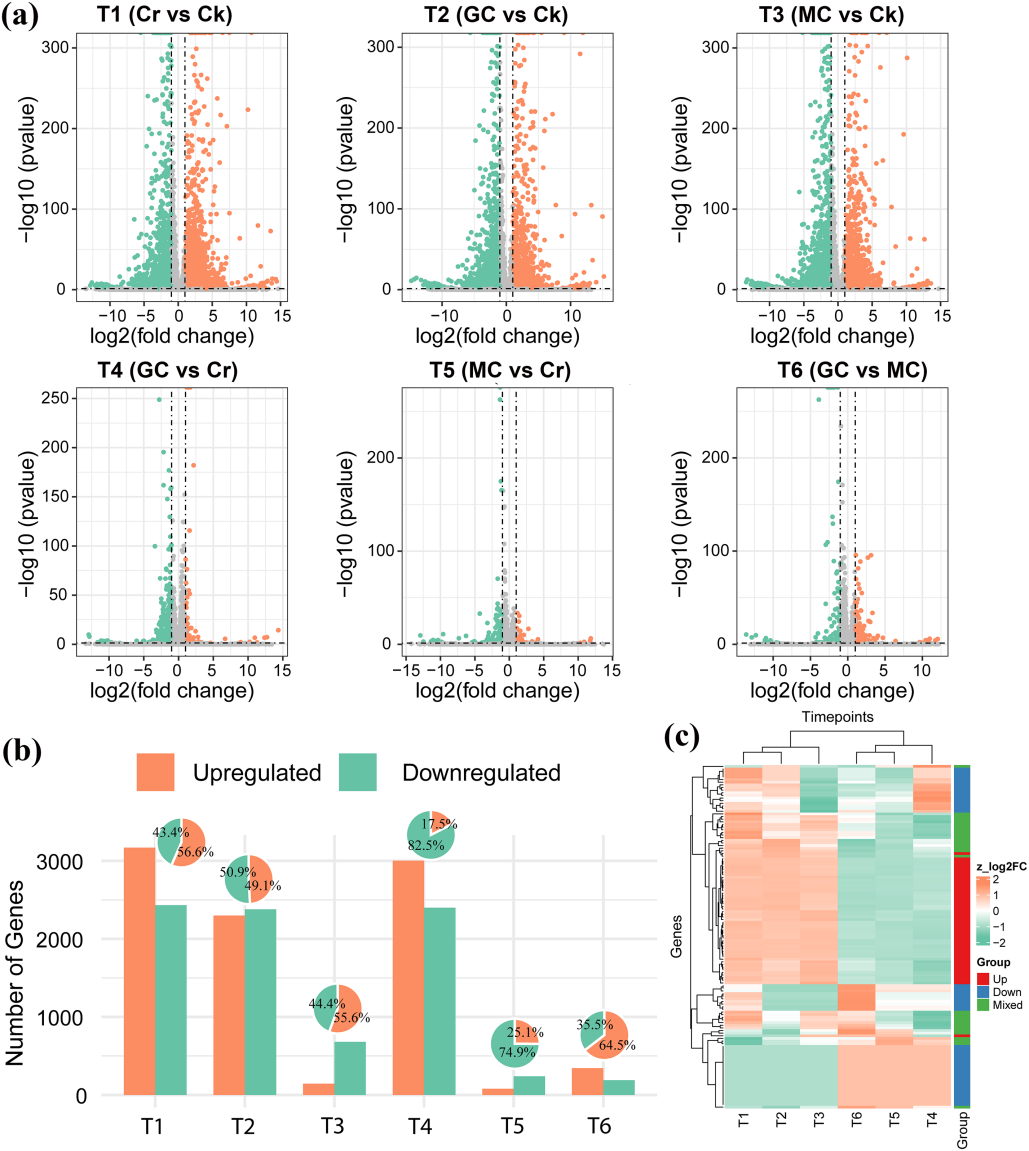
Identification and expression patterns of differentially expressed genes (DEGs) across chromium stress and protectant treatments. This figure summarizes the DEG counts, their regulation trends, and the expression profiles of the most significantly regulated genes in the six pairwise comparisons. **(a)** Volcano plots for pairwise comparisons (T1 to T6). **(b)** Column plots showing the number of up- and downregulated DEGs. **(c)** Heatmap displays the normalized expression profiles of the most significant DEGs across all treatment groups.

Using a threshold of log_2_FC ≥ 1 and FDR ≤ 0.05, Cr stress versus CK (T1) yielded 5,601 DEGs, of which 56.60% were upregulated, and 43.40% were downregulated (**Figures 2a, b**; **Supplementary Table 3**). In plants applied with GSH under Cr (T2: GC vs CK), 4,677 DEGs were detected, with a nearly balanced distribution of 49.10% upregulated and 50.90% downregulated genes. MT under Cr (T3: MC vs CK) produced 5,401 DEGs, with 55.60% upregulated and 44.4% downregulated, indicating a transcriptional response similar in magnitude to Cr alone (**Figures 2a, b**). Direct comparisons with Cr alone revealed that GSH (T4: GC vs Cr) reprogrammed 824 genes, with a strong bias toward repression (17.50% up- vs 82.50% downregulated), whereas MT (T5: MC vs Cr) affected only 319 genes (25.10% up- and 74.90% downregulated). The GC vs MC contrast (T6) identified 533 DEGs, of which 64.50% were higher in GC, and 35.50% were lower. These DEG patterns are consistent with our hypothesis that although both MT and GSH act in a Cr-stress context, GSH exerts a quantitatively stronger and more distinct modulation of the Cr-responsive transcriptome than MT (**Figures 2a–c**). The top upregulated and downregulated genes showed different expression profiles under different groups (**Figures 2a–c**).

### 3.3 Biological functions of differentially expressed genes

**Figure 3:**
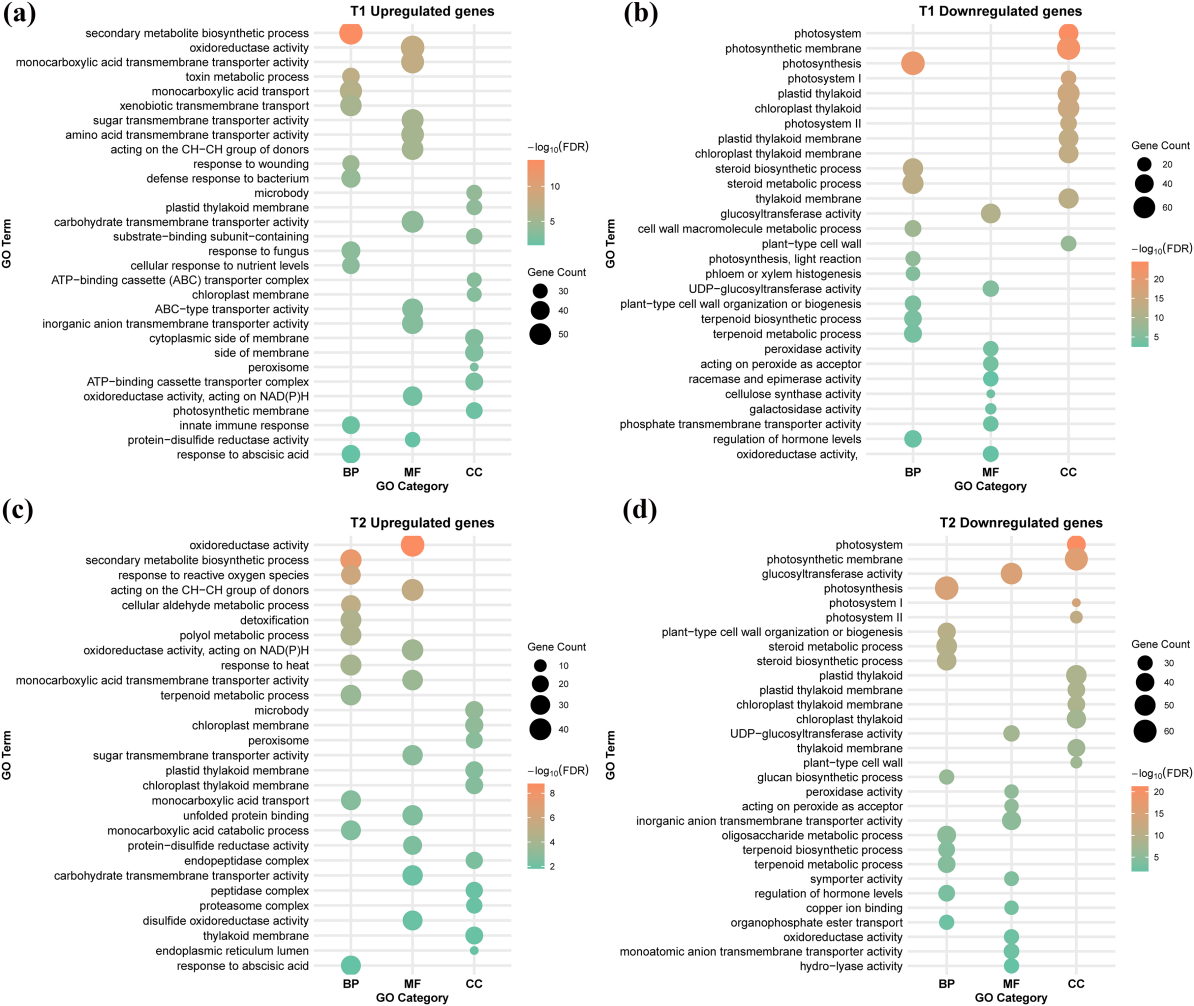
Gene Ontology (GO) enrichment analysis of differentially expressed genes (DEGs) under Cr stress and GSH mitigation. **(a–d)** The figure presents the most significantly enriched biological process (BP), cellular component (CC), and molecular function (MF) GO terms for the two main comparisons: Cr stress vs. Control (T1) and GSH mitigation vs. Cr stress (T2).

GO enrichment analysis of DEGs (FDR ≤ 0.05) for the three treatments (T1 to T3) versus CK showed a common pattern of activation of detoxification/transport processes and repression of photosynthetic and growth-related functions (**Figure 3**; **Supplementary Figure S1**; **Supplementary Table 4**). In T1 (Cr vs CK), upregulated genes were mainly involved in xenobiotic transmembrane transport, toxin and secondary-metabolite metabolism, responses to wounding, fungus and abscisic acid, monocarboxylic acid transport, and cellular nutrient responses (**Figure 3a**). These genes were enriched in peroxisomes, chloroplasts, and other membranes, and ABC transporter complexes, with molecular functions dominated by various transmembrane transporters and oxidoreductase activities. Downregulated genes in T1 were strongly enriched in steroid/terpenoid biosynthesis and metabolism, regulation of hormone levels, cell-wall macromolecule metabolism/organization, and photosynthesis and light reactions, together with chloroplast and thylakoid membrane terms and peroxidase/glucosyltransferase activities (**Figure 3b**). Likewise, in T2 (GC vs CK), the upregulated DEGs showed a similar but more detoxification-focused profile, with significant enrichment of response to ROS, cellular aldehyde metabolic process, detoxification, terpenoid metabolism, ABA response, monocarboxylic acid transport/catabolism, and polyol metabolism (**Figure 3c**). In contrast, downregulated GC-responsive genes were enriched in steroid/terpenoid and glucan/oligosaccharide biosynthesis, cell-wall organization, photosynthesis, and photosynthetic membrane components, indicating that photosynthetic machinery remains lower than in CK despite GSH treatment (**Figure 3d**). In T3 (MC vs CK), upregulated genes were enriched for xenobiotic metabolism and transport, toxin metabolism, and transporter/oxidoreductase activities associated with peroxisomes, and ABC transporter complexes (**Supplementary Figure 2**). In contrast, downregulated genes showed the same core categories as T1 and T2; steroid/terpenoid biosynthesis, photosynthesis and photosynthetic membranes, and glucan/cell-wall metabolism (**Supplementary Figure 2**). These results indicate that the shared GO signatures confirm that Cr stress suppresses chloroplast and primary metabolic functions. However, GSH particularly strengthens ROS and aldehyde detoxification pathways compared with Cr alone and MT (**Figure 3**; **Supplementary Figure S2**).

In the direct comparisons under Cr stress, GO enrichment highlighted that GSH and MT differentially remodel the stress transcriptome (**Supplementary Figures 3**, **4**). Relative to Cr alone (T4), GC upregulated genes associated with peptidase regulation and inhibitor activity, as well as acetyl-/acyltransferases and glyoxalase enzymes, indicating enhanced protein quality control and methylglyoxal/ROS detoxification. Conversely, GC downregulated genes related to cell-wall formation, apoplast/cell-surface signaling, and solute symport, consistent with a reduced need for cell-wall remodeling and stress signaling once Cr toxicity is alleviated. Compared with Cr (T5), MC mainly induced genes involved in membrane and vesicle transport and metal/amine ion transport, while repressing amino-acid, nitrogen, and sulfur compound biosynthesis. This suggests that MT adjusts intracellular trafficking and secondary metabolism but invests less in sulfur-based detox pathways than GC. The GC vs MC contrast (T6) further showed that GC preferentially upregulated ion and anion homeostasis, including nitrite efflux and inorganic anion transport. In contrast, genes linked to carbohydrate/biogenic amine metabolism and proteasome/peptidase regulation were relatively higher in MC. These GO patterns support the view that GSH more strongly reinforces redox and detoxification networks and ion homeostasis than MT under the same Cr stress (**Supplementary Figures 2**, **3**).

### 3.4 Differentially expressed genes involved in metabolic pathways

**Figure 4:**
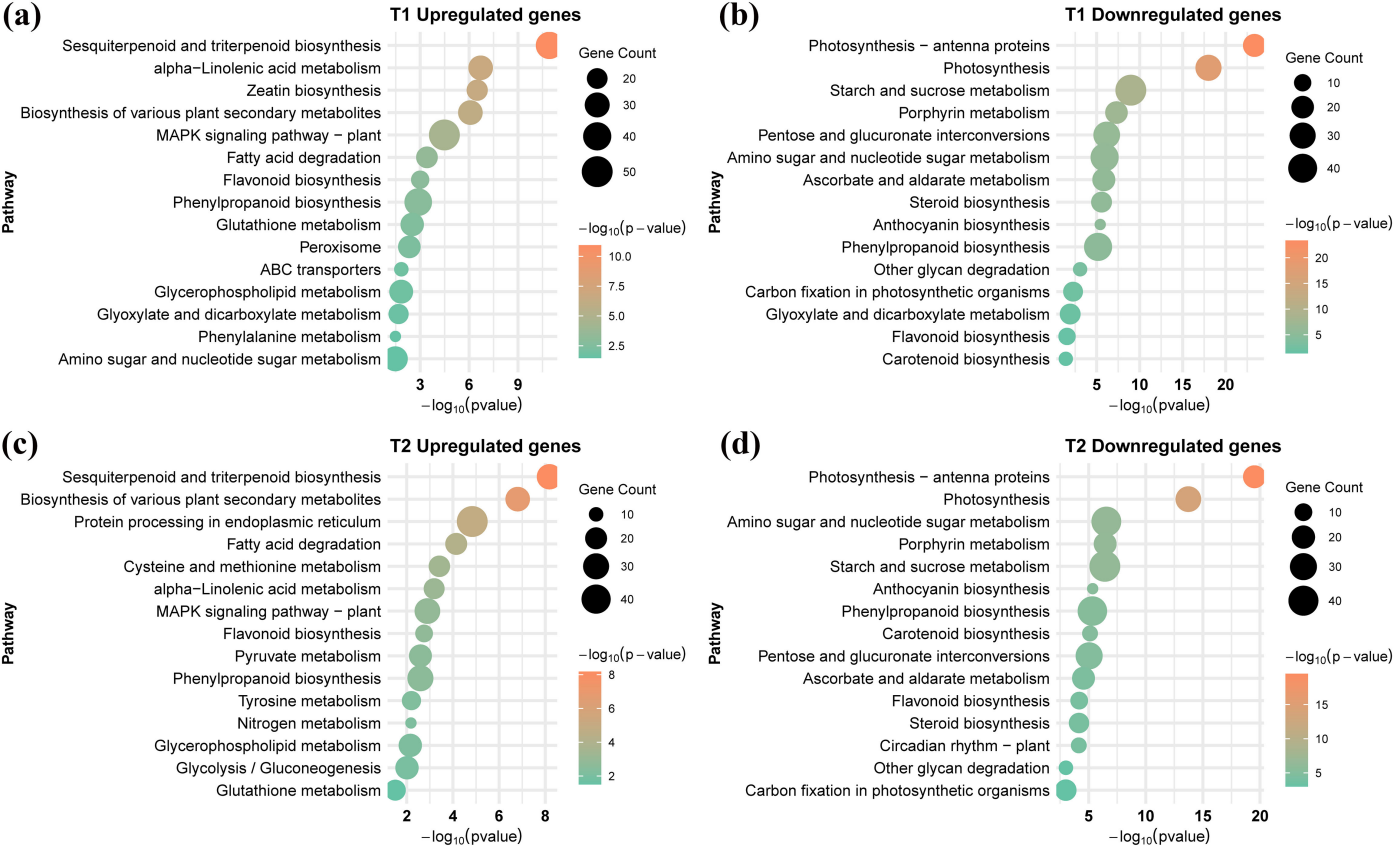
KEGG pathway enrichment analysis of DEGs under Cr stress and GSH mitigation. **(a–d)** The figure presents the most significantly enriched KEGG pathways for the two primary comparisons: Cr stress vs. Control (T1) and GSH mitigation vs. Cr stress (T2).

KEGG enrichment of DEGs (FDR ≤ 0.05) for T1-T3 versus CK showed that Cr stress mainly activated stress-responsive lipid and secondary metabolism while suppressing photosynthetic carbon metabolism (**Figure 4**; **Supplementary Figure S3**; **Supplementary Table 5**). In T1 (Cr vs CK), upregulated genes were enriched in sesquiterpenoid and triterpenoid biosynthesis, α-linolenic acid metabolism, zeatin and other secondary-metabolite biosynthesis, MAPK signaling, glutathione metabolism, and peroxisome pathways, consistent with enhanced hormone/oxylipin signaling and ROS detoxification. Conversely, Cr strongly downregulated photosynthesis and antenna proteins, starch and sucrose metabolism, carbon fixation, porphyrin and carotenoid biosynthesis, and several sugar and steroid biosynthetic pathways, reflecting inhibition of light harvesting and primary carbon metabolism. In T2 (GC vs CK) and T3 (MC vs CK), upregulated pathways largely overlapped with the Cr response, including terpenoid and flavonoid/phenylpropanoid biosynthesis, α-linolenic acid metabolism, fatty-acid degradation, and MAPK signaling. However, GC uniquely enriched cysteine and methionine metabolism, glycolysis/gluconeogenesis, pyruvate and nitrogen metabolism, and protein processing in the ER, together with glutathione metabolism, indicating that exogenous GSH more strongly reinforces sulfur assimilation, central carbon metabolism, and the GSH pool required for detoxification.

In the direct comparisons under Cr stress, KEGG enrichment highlighted apparent differences between GSH and MT responses (**Supplementary Figures 3**, **4**). Relative to Cr alone (T4), GSH-upregulated genes were mainly enriched in glutathione metabolism, cysteine and methionine metabolism, and related carbon/nitrogen metabolism pathways, consistent with reinforced sulfur-based antioxidant capacity and detoxification. Pathways reduced by GC included several cell wall- and stress-signaling-related routes, in line with a reduced stress load once Cr toxicity is alleviated. In contrast, MT vs Cr (T5) mainly enriched pathways associated with plant hormone signal transduction, MAPK signaling, and membrane/vesicle transport, suggesting that MT primarily fine-tunes signaling and trafficking rather than strongly boosting core detox metabolism. The GC vs MC contrast (T6) further showed that genes higher in GC were enriched in glutathione and sulfur metabolism, nitrogen metabolism, and other redox-related pathways. In contrast, genes higher in MC were more associated with secondary metabolism and protein processing.

### 3.5 Cluster analysis identified glutathione-associated genes

**Figure 5:**
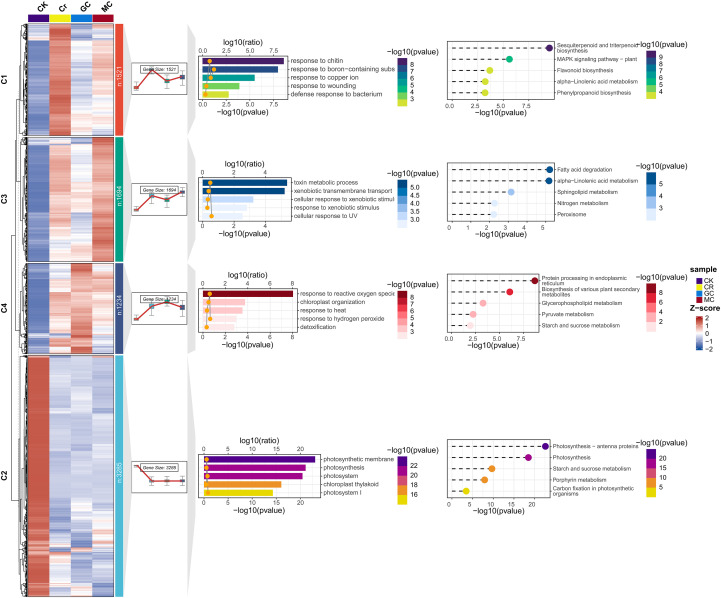
Clustering analysis of all DEGs and associated functional enrichment. The figure provides a mechanistic framework by grouping the DEGs into four major expression clusters (C1 to C4) based on their coordinated response across CK, Cr, GC, and MC treatments, showing the GO terms and KEGG pathways associated with each functional module.

To obtain a mechanistic view of the transcriptomic response, we clustered all DEGs across the six comparisons, yielding 7,734 genes that were grouped into four major expression clusters (C1–C4) (**Figure 5**; **Supplementary Table 6**). Cluster sizes ranged from 1,234–3,285 genes, with C1 containing 1,521 genes (19.7%), C3 containing 1,694 genes (21.9%), C4 containing 1,234 genes (16.0%), and C2 containing the largest group with 3,285 genes (42.5%). The z-score heatmap and average expression profiles clearly separated Cr stress (CR) from the control (CK) and revealed distinct GC- and MC-modulated patterns under the same Cr background. C1 comprised genes strongly induced by Cr relative to CK, whose expression was partially reversed by GC and, to a slight extent, by MC. GC brought these Cr-upregulated genes back to basal levels, whereas MC maintained a moderate induction, indicating that GC suppresses a larger subset of Cr-activated transcripts more efficiently. C3 also contained Cr-induced genes, but MC further enhanced their expression beyond Cr, while GC slightly reduced their levels. This pattern defines an MC-preferential module, suggesting that MT tends to amplify specific Cr-responsive pathways rather than reduce them. C4 represented another Cr-induced group in which GC further boosted expression above Cr, with MC showing intermediate induction. These genes likely correspond to GC-enhanced detoxification and defense programs that are selectively activated in the presence of GSH. In contrast, C2 genes were highly expressed in CK but strongly repressed by Cr, and remained low under both GC and MC treatments, indicating a large cohort of Cr-suppressed, putatively growth-associated genes that neither treatment fully restores. The clustering analysis shows that, although both GC and MC act on Cr-responsive genes, GSH predominantly counteracts harmful Cr-induced expression (C1) and reinforces a specific set of protective genes (C4). In contrast, MT more often maintains or even strengthens Cr-like expression states (C3). These coordinated expression modules provide a mechanistic framework supporting GSH as the more effective regulator of the Cr-stress.

Consistent with these expression patterns, GO and KEGG enrichment confirmed that each cluster represents a functionally coherent module (**Figure 5**). C1 was enriched for stress- and defense-related GO terms such as response to wounding, chitin, bacteria, and metal ions, together with KEGG pathways including α-linolenic acid metabolism, sesquiterpenoid/triterpenoid, phenylpropanoid, and flavonoid biosynthesis, and MAPK signaling pathway, indicating a broad Cr-induced defense program that is reduced mainly by GC. C2 was strongly enriched for photosystem and chloroplast thylakoid components and for photosynthesis-related processes, with KEGG terms including photosynthesis, antenna proteins, starch and sucrose metabolism, carbon fixation, and porphyrin metabolism, consistent with a core photosynthetic module that is repressed by Cr and not fully restored by either treatment. C3 showed GO enrichment for xenobiotic transport and toxin/xenobiotic metabolism, and KEGG enrichment for fatty-acid degradation, α-linolenic acid and sphingolipid metabolism, nitrogen metabolism, and peroxisome, defining an MC-preferential detoxification and lipid–nitrogen remodeling module. Finally, C4 was enriched for responses to ROS, H_2_O_2_, heat, and chloroplast organization, with KEGG pathways such as starch and sucrose metabolism, glycerophospholipid and pyruvate metabolism, secondary-metabolite biosynthesis, and protein processing in the ER, highlighting a GC-enhanced module that combines redox detoxification with metabolic and protein-quality control adjustments under Cr stress.

### 3.6 Identification of modules associated with physio-chemical traits

**Figure 6:**
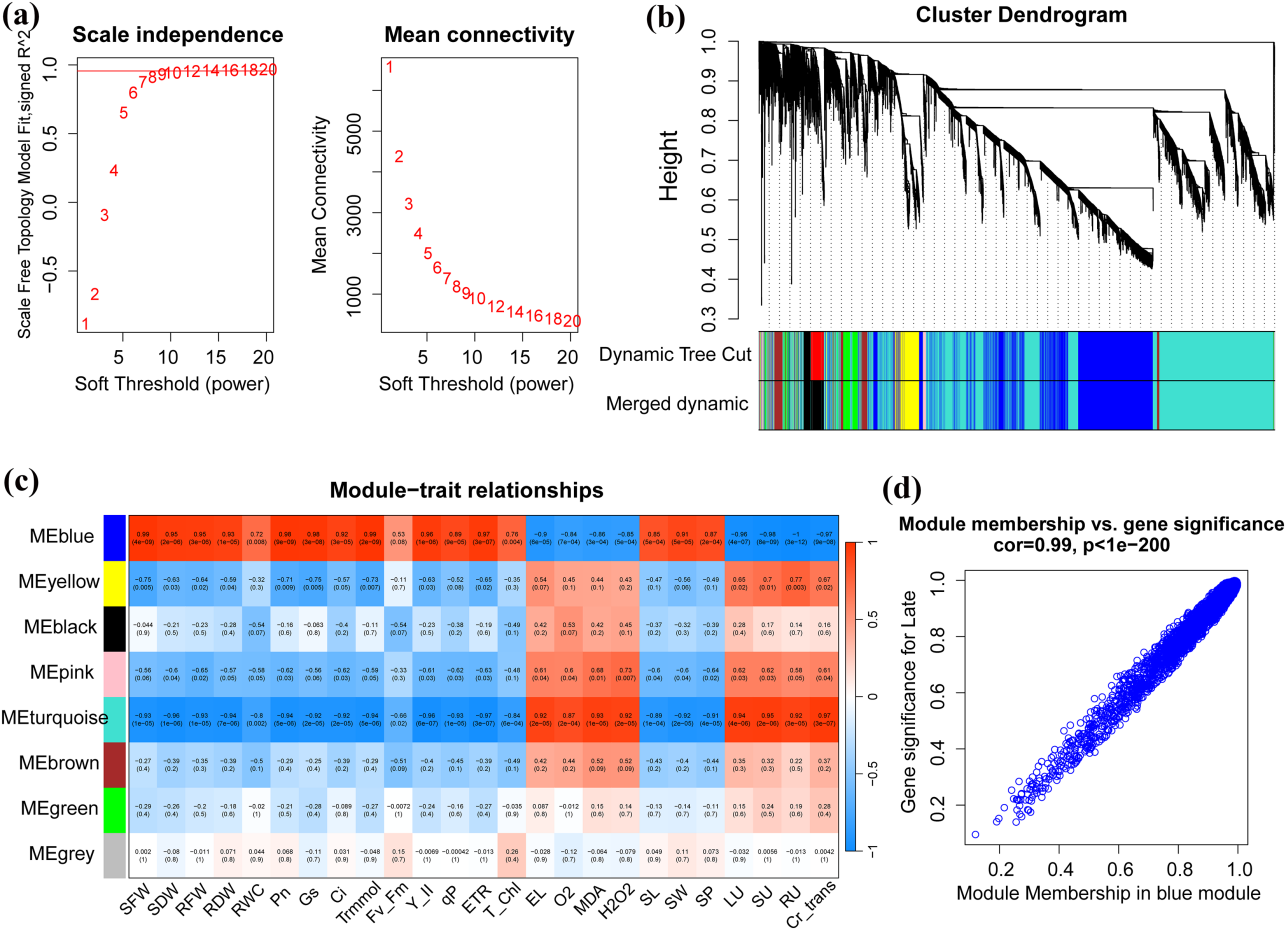
Weighted Gene Co-expression Network Analysis (WGCNA) for identifying co-expressed gene modules related to Cr stress and mitigation. **(a)** Analysis of network topology for soft-threshold power, scale-free topology fit index (R2). **(b)** Hierarchical cluster dendrogram of genes, which displays the clustering of all genes based on the topological overlap measure (TOM), where each major branch or leaf constitutes a co-expressed gene module, color-coded for visual distinction. **(c)** Pearson correlation coefficient and associated P-values between the gene modules (rows) and the four experimental conditions treated as traits. **(d)** Scatter plot of gene significance (GS) vs. module membership (MM), which visualizes the strong correlation between GS and MM for the module.

To further dissect the coordinated transcriptional responses underlying Cr detoxification by GSH and MT, we constructed a co-expression network using the top 25% most variable genes. A soft-thresholding power of β = 12 produced a scale-free topology with R^2^ = 0.90, indicating a biologically meaningful network structure (**Figure 6a**). WGCNA grouped the genes into seven co-expression modules with dynamic tree-cut (blue, yellow, black, pink, turquoise, brown, green, and grey), and their eigengenes were correlated with growth, photosynthetic, oxidative-stress, and Cr-accumulation traits (**Figure 6b**). Among these, the blue module showed the most distinct pattern and its eigengene was strongly and positively correlated with biomass and water-status traits (SFW, SDW, RFW, RDW, and RWC; *r* = 0.72-0.99, *p* ≤ 0.01) and with photosynthetic performance (Pn, Gs, Ci, Trmmol, Y(II), qP, ETR, and total chlorophyll; *r* = 0.76-0.99, generally *p* < 10^-3^) (**Figures 6c, d**). In contrast, the blue eigengene was negatively correlated with membrane injury and oxidative markers (EL, O_2_^-^, MDA, and H_2_O_2_; *r* = -0.84 to -0.90, *p* ≤ 10^-3^) and showed robust negative correlations with Cr accumulation and translocation parameters (LU, SU, RU, and Cr_trans; r = −0.98 to −1.00, p ≤ 10^-7^). Modules such as yellow and turquoise displayed an opposite trend, as they were negatively associated with growth and photosynthetic traits and positively associated with oxidative damage and Cr accumulation, suggesting that these modules represent Cr-sensitive expression programs. These results highlighted that the blue module is a core Cr-tolerance module and upregulation is associated with improved growth, photosynthetic efficiency, and reduced Cr uptake/translocation. Therefore, these physiological improvements are most pronounced under GSH treatment; the blue module likely captures a significant portion of the GC-driven protective network against Cr stress.

### 3.7 Mining of candidate hub genes associated with physio-chemical trait improvement

**Figure 7:**
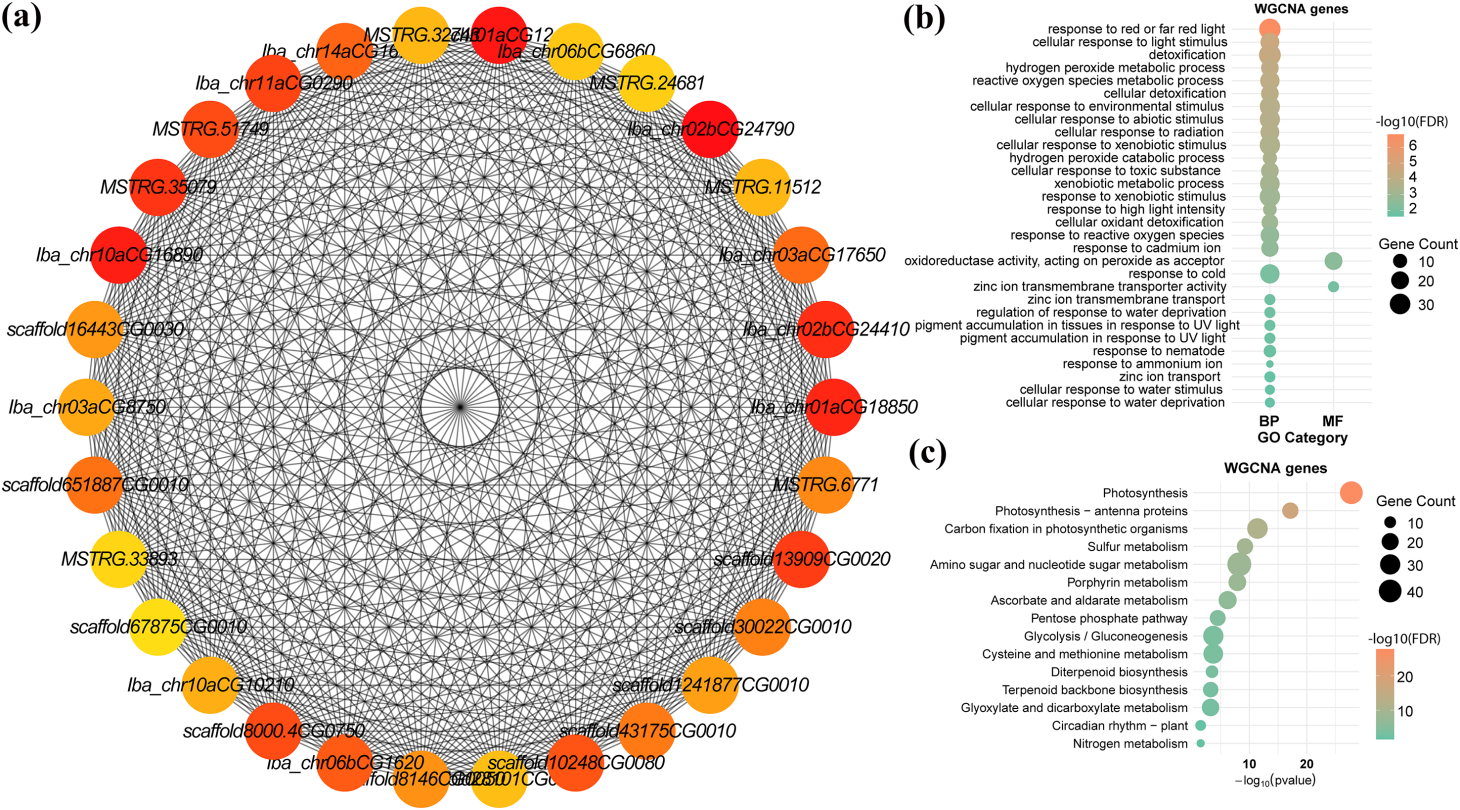
Visualization and functional analysis of key co-expressed hub genes associated with the Cr stress and GSH and MT mitigation response in sweet potato. **(a)** Visualization of the co-expression subnetwork represents the top 30 hub genes from the highly significant blue module, shown as nodes, with edges representing the strong co-expression relationships between these genes. **(b, c)** GO and KEGG enrichment analysis of the blue module hub genes.

Following WGCNA analysis, we selected the top 30 hub genes by degree as key candidates underlying the physio-chemical improvements observed in GSH-treated plants (**Figure 7**; **Supplementary Table 7**). Several hub genes, such as *HEMA1, CHLP, CAB4, CAB13, PSAN*, and *PSAL*, encode enzymes and structural proteins involved in chlorophyll/tetrapyrrole biosynthesis and photosystem I light harvesting, directly linking the GC module to the recovery of chlorophyll content, Fv/Fm, and electron transport efficiency under Cr stress. A second group of hubs is closely tied to redox and detoxification processes that cooperate with glutathione metabolism, including a peroxidase annotated with “response to oxidative stress,” “hydrogen peroxide catabolic process,” and phenylpropanoid biosynthesis, and *CHLP/HEMA1* with GO terms related to cell redox homeostasis and porphyrin metabolism. These genes likely work in concert with the AsA-GSH cycle to remove ROS and toxic compounds in GC-treated plants. Transport- and osmotic-regulation hub genes, such as aquaporin *TIP2-1* (two copies) and the phosphate transporter *PHT1-4*, together with *TPPA* (trehalose-phosphate phosphatase), are associated with improved water status, nutrient uptake, and osmoprotectant (trehalose) metabolism, consistent with higher RWC, better root architecture, and reduced EL. Growth, signaling, and structural adjustment are represented by hub genes, such as *GA20OX1/GA20OX2* (gibberellin biosynthesis), the HD-ZIP transcription factor *HAT4* (light and hormone-responsive developmental regulator), a receptor-like serine/threonine kinase, and multiple cell-wall and lipid–related enzymes (*XTH1*, pectate lyases, alpha-mannosidase, *ASAT1*, and *CAS1*). These genes integrate hormone signaling, cell-wall remodeling, and membrane/lipid homeostasis with GSH-enhanced detoxification to support biomass recovery and cell integrity under Cr. These 30 candidate hub genes, which are directly or indirectly associated with enhanced photosynthesis, antioxidant capacity, osmotic balance, and growth under Cr stress with mitigator treatment, represent promising targets for sweet potato breeding programs and genetic engineering aimed at improving chromium stress tolerance.

### 3.8 Validation of RNA-seq via RT-qPCR analysis

**Figure 8:**
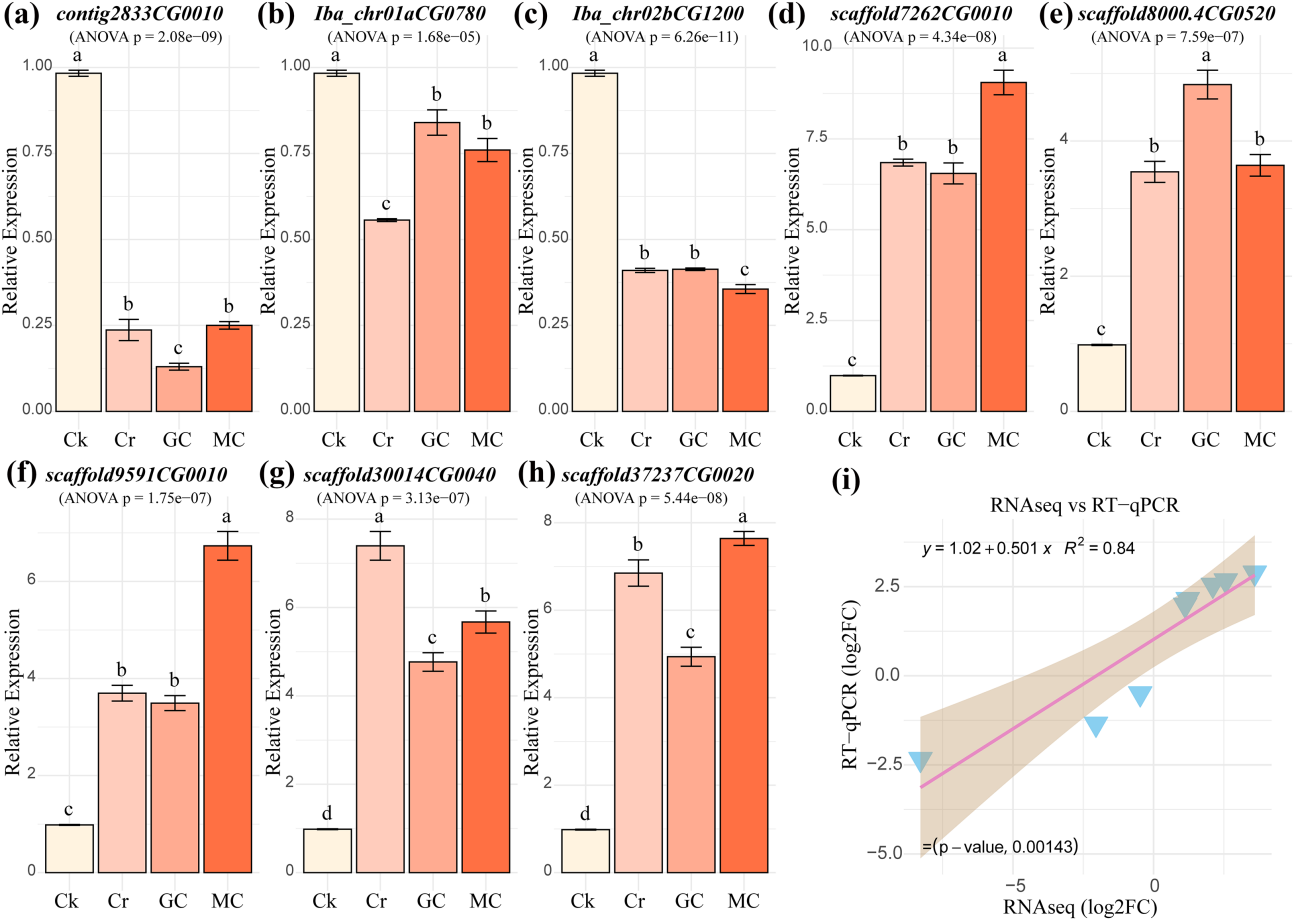
Validation of RNA-seq data using quantitative reverse transcription polymerase chain reaction (RT-qPCR) analysis. **(a–h)** Relative expression levels of eight candidate genes. Bar plots showing the mean relative expression of the eight selected genes, with different letters between the bars indicating significance at p ≤ 0.05. **(i)** A scatter plot demonstrating the linear relationship between the Log2FC values obtained from RNA-seq and RT-qPCR for the eight selected genes, with the Pearson correlation coefficient clearly displayed, validating the consistency of the transcriptome dataset.

To validate the reliability and consistency of the RNA-seq data, the expression levels of eight candidate genes were analyzed by RT-qPCR using the same CK, Cr, GC, and MC samples (**Figure 8**). The RT-qPCR expression patterns further corroborated the study’s key functional outcomes. Genes involved in metal transport, such as the metal transporter *NRAMP5* (*contig2833CG0010*), were strongly repressed by Cr (~0.24), and this repression was further enhanced by GSH (~0.13), suggesting that GSH assists the plant in actively limiting Cr uptake. Conversely, genes central to defense and signaling were significantly induced by Cr (> 3.50). The expression of the GST enzyme *HSP26-A* (*scaffold37237CG0020*), which is crucial for detoxification, was highly induced by Cr (~6.85) but was notably reduced by GSH treatment (~4.94). This confirms that GSH alleviates the internal oxidative stress load, thereby reducing the necessity for massive GST transcription, whereas MC maintained a high level of induction (~7.64). The transcription factor *WRKY70* (*scaffold8000.4CG0520*) was induced by Cr (~3.54), but its expression was further enhanced by GSH (~4.83), aligning with the finding that GSH actively reinforces stress signaling and defense programs. Furthermore, the MT treatment specifically showed the highest induction for the transcription factor *ERF113* (*scaffold7262CG0010*, approximately 9.05) and the receptor GLR1.3 (~6.73), supporting the role of MT in fine-tuning specific hormone and signaling pathways, as previously indicated by the cluster analysis. These RT-qPCR results collectively validate the RNA-seq dataset and provide a specific functional context for the GSH- and MT-mediated detoxification strategies observed. The correlation between the Log2FC values obtained from the RNA-seq analysis and the RT-qPCR assay was calculated (**Figure 8i**). The results showed a strong positive Pearson correlation coefficient (r) of 0.915 across the eight selected genes, indicating a high degree of concordance between the two independent quantification methods and robust validation of the transcriptome analysis.

## 4 Discussion

Sweet potato is a nutritionally rich staple and cash crop, but, like many other crops, it is highly vulnerable to yield and quality losses due to HM contamination. In particular, Cr disrupts photosynthesis, root growth, and redox homeostasis and poses direct health risks when it accumulates in edible tissues. In recent years, MT and GSH have attracted attention as practical tools to enhance Cr tolerance. Therefore, it is urgent to identify candidate genes that confer HM tolerance and could be useful for future breeding programs. In this study, we used RNA-seq to systematically compare CK, Cr, MC, and GC samples, which provide a crosstalk mechanism between Cr stress, MC, and GC. As a result, we identified 7,734 DEGs across six contrasts, four major expression clusters (C1–C4) that distinguish Cr-, MT-, and GSH-responsive modules, and 30 candidate hub genes tightly associated with improvements in photosynthesis, redox balance, osmotic adjustment, and growth. These results indicate that GSH has an advantage over MT in mitigating HM stress via detoxification, redox homeostasis, cell wall modification, and signaling pathways.

The successful decoding of the molecular response to Cr stress in this study, supported by high-quality sequencing metrics and robust alignment statistics, provides a high-resolution view of the comparative roles of exogenous GSH and MT in conferring tolerance. Our foundational hypothesis, partially supported by previous physiological investigations (Dorion et al., 2021; Kumar et al., 2024), proposed that both molecules mitigate Cr toxicity; GSH might induce a more potent and distinct transcriptional shift than MT. The differential expression analysis further substantiated the distinct roles of the two alleviators, confirming GSH as the stronger transcriptional modulator. Cr stress alone vs CK generated 5,601 DEGs, and MT application produced a similar 5,401 DEGs. The direct comparison against the stressed condition reveals the key difference. GSH application reprogrammed 824 genes, which is approximately 2.6 times more than the 319 genes induced by MT application. This quantitative superiority of GSH at the transcriptional level aligns perfectly with our prior physiological findings, which demonstrated that GSH was generally more effective than MT in enhancing growth, improving photosynthetic performance, and mitigating oxidative damage under Cr stress (Kumar et al., 2024). Crucially, the directional bias of the GC vs Cr comparison provides a mechanistic clue: 82.50% of the 824 DEGs were downregulated, which indicates a strong bias toward transcriptional repression, suggesting that the GSH-driven detoxification strategy involves the shutting down of non-essential or resource-intensive pathways and the fine-tuning of energy reallocation to maximize tolerance (Manzoor et al., 2021; Chen et al., 2022). Conversely, the MC vs Cr contrast also showed a repressive bias (74.90% downregulated DEGs), but on a much smaller scale. The GC vs MC comparison, which yielded 533 DEGs with a bias toward higher expression in GC (64.50%), highlights that the GSH-response involves the unique upregulation of a core set of detoxification genes that MT does not activate. These findings are consistent with the well-established role of GSH as a central hub in plant defense: It not only acts as a critical antioxidant, but also serves as a precursor for PCs chelation and a fundamental component of the AsA-GSH cycle (Zhou et al., 2018; Yang et al., 2023). The induction of pathways like glutathione metabolism is a common and crucial feature in heavy metal tolerance across various species (Zhou et al., 2023; Liu et al., 2024). While MT is known to enhance the GSH antioxidant system under stress (Shi et al., 2015; Li et al., 2022), our data suggest that exogenous GSH bypasses and intensifies this regulatory loop, directly forcing the cell into a more efficient, repressed, detoxification state, consistent with the observed separation from Cr and MC in the PCA.

GO and KEGG pathway enrichment analyses provided molecular confirmation of the stress response and the divergent actions of the protectants (**Figures 3**, **4**). The common transcriptional signature across all Cr-stressed samples (T1-T3 vs CK) was the strong repression of primary metabolic and growth-related processes, including photosynthesis, carbon fixation, starch and sucrose metabolism, and cell-wall organization (Yang et al., 2023; Zhou et al., 2023). Simultaneously, Cr induced core plant defense mechanisms, notably enriching pathways for xenobiotic and toxin metabolism, MAPK signaling, and glutathione metabolism, which is consistent with established responses to HM toxicity (Cui et al., 2020) and biotic stress (Farhan et al., 2025b, a). However, the protective agents induced distinct detoxification programs: while MT (MC vs Cr) primarily modulated pathways linked to plant hormone signal transduction and membrane/vesicle transport, suggesting a focus on signaling fine-tuning and trafficking (Shi et al., 2015). In contrast, exogenous GSH (GC vs Cr) uniquely and significantly reinforced the central detox machinery. This GSH-specific response enriched cysteine and methionine metabolism, glyoxalase activity, and central carbon/nitrogen metabolism, confirming that GSH acts to directly boost the sulfur assimilation required for PC synthesis and the aldehyde detoxification system (Cui et al., 2020; Zhu et al., 2022). These results validate the conclusion that GSH is a stronger transcriptional re-programmer, shifting the transcriptome toward a uniquely reinforced redox-centric, detoxification-dominant metabolic state.

We identified 30 candidate genes through WGCNA analysis, which are associated with biomass, water-status traits, photosynthetic traits, membrane injury and oxidative markers, and Cr accumulation and translocation parameters. In addition, these 30 genes overlapped with DEGs, significantly validating their functional relevance and confirming that they are not merely correlated with the phenotype but are also strongly and actively regulated at the transcriptional level in response to Cr, GSH, and MT treatments. Therefore, these genes are the most robust and highly confident candidates for regulating the sweet potato’s Cr detoxification network. The repression of essential growth processes is evidenced by the predicted downregulation of *Iba_chr02bCG24790* (*GA20OX2*) and *MSTRG.35079* (*GA20OX1*), which encodes gibberellin 20 oxidase 1-like enzymes and regulates the biosynthesis of active gibberellins (Zhang et al., 2023b). The downregulation of these genes aligns with the observed suppression of plant growth and the plant’s strategy to conserve resources under HM toxicity (Maleki et al., 2017; Huihui et al., 2020). The inhibition of the light-dependent machinery further highlights this metabolic shift away from anabolism, specifically *Iba_chr01aCG18850* (*HEMA1*), which encodes glutamyl-tRNA reductase 1, the rate-limiting enzyme in chlorophyll synthesis (McCormac and Terry, 2002; Ayyaz et al., 2021), and the core photosystem components *CAB4* and *PSAN*, which encode chlorophyll a-b binding protein and photosystem I reaction center subunit N, respectively, confirming the disruption of photosynthetic carbon assimilation detailed in the GO and KEGG analyses (Zhou et al., 2023; Zheng et al., 2024). In contrast, genes associated with stress response mechanisms showed regulation necessary for Cr tolerance. Gene *scaffold8000.4CG0750* encodes a peroxidase 4-like enzyme that plays a crucial role in the ROS scavenging network, and its potential upregulation is a direct countermeasure against oxidative stress caused by Cr exposure (You and Chan, 2015; Caverzan et al., 2016). Furthermore, two genes, *Iba_chr10aCG16890* (*At4g26220*) and *scaffold10248CG0080* (*At4g26220*), encode caffeoyl-CoA O-methyltransferase, an enzyme critical for lignin biosynthesis. The activation of this enzyme promotes cell wall rigidification, serves as a structural barrier to Cr sequestration, and enhances defense responses, correlating with the overall activation of secondary metabolism pathways (Zhao et al., 2021; Luan et al., 2023). Finally, genes such as *Iba_chr01aCG12400* (*TIP2-1*), which encodes an aquaporin TIP2-1-like protein, may regulate water and solute transport across the tonoplast, potentially facilitating the vacuolar sequestration of toxic heavy metal ions or their chelate complexes (Song et al., 2016; Vishwakarma et al., 2019; Zhang et al., 2023a). These hub genes represent key nodes that inhibited growth/photosynthesis and induced detoxification/barrier formation, thereby governing sweet potato’s response to Cr stress. However, these genes require functional validation to determine their exact roles in HM stress.

## 5 Conclusion

This study successfully elucidated the mitigating roles of MT and GSH in alleviating the adverse effects of Cr stress in sweet potato, providing integrated insights across physiological, biochemical, and transcriptional levels. Our findings confirm that Cr toxicity induces a core stress signature characterized by severe inhibition of growth and photosynthetic capacity, coupled with the activation of fundamental detoxification and oxidative defense mechanisms. Crucially, the comparative analysis depicted that GSH provided a superior protective effect compared to MT, particularly in restoring photosynthetic efficiency and reducing Cr accumulation. GO and KEGG enrichment results, validated by the identification of 30 overlapping DEA hub genes, provided the molecular basis for this distinction. The Cr stress response in all treatments involved the coordinated suppression of primary metabolism (photosynthesis and GA biosynthesis) and the activation of secondary metabolism (terpenoid, lignin, and xenobiotic metabolism). GSH treatment uniquely and significantly amplified this defense network, predominantly through the transcriptional reinforcement of sulfur assimilation (cysteine and methionine metabolism) and glutathione metabolism. These findings suggest that GSH directly boosts the plant’s capacity to synthesize PCs and enhances ROS scavenging, thereby providing a more direct and effective systemic detoxification mechanism. GSH reconfigures core central and sulfur-based metabolism to sustain the cellular redox balance and enhance detoxification, while MT focuses on fine-tuning stress signaling and cellular trafficking. These hub genes and pathways represent high-confidence targets for future functional genomics studies and offer promising molecular markers for developing Cr-tolerant sweet potato cultivars through targeted breeding or genetic engineering.

## Data Availability

The original contributions presented in the study are publicly available. This data can be found here: https://ngdc.cncb.ac.cn/gsa/browse/CRA038896.

## Supplementary material

Supplementary Figure 1 Gene Ontology (GO) enrichment analysis of Differentially Expressed Genes (DEGs) for MT mitigation and the direct GSH vs. MT comparison.

Supplementary Figure 2 Gene Ontology (GO) enrichment analysis of DEGs showing the overall effect of MT and GSH on the transcriptome relative to the CK.

Supplementary Figure 3 KEGG pathway enrichment analysis of DEGs for MT mitigation and the direct GSH vs. MT comparison.

Supplementary Figure 4 KEGG pathway enrichment analysis of DEGs showing the overall effect of MT and GSH on the transcriptome relative to the CK.

Supplementary Table 1 List of primers and their sequences used in RT-qPCR analyses.

Supplementary Table 2 Read mapping rates aligned to reference genome of sweet potato.

Supplementary Table 3 List of identified DEGs under Cr stress.

Supplementary Table 4 The GO enrichment analysis of upregulated and downregulated differentially expressed genes.

Supplementary Table 5 KEGG enrichment analysis of upregulated and downregulated differentially expressed genes.

Supplementary Table 6 Cluster analysis of differentially expressed genes.

Supplementary Table 7 List of candidate hub genes involved in Cr stress tolerance in sweet potato.
