# Supplementary figures and images for: Crosstalk between glutathione and melatonin in chromium detoxification in sweet potato revealed by high-throughput sequencing and physio-biochemical profiling
Source: Front Plant Sci. 2026 Mar 2;17:1767742. doi: 10.3389/fpls.2026.1767742 (PMC12989550; doi:10.3389/fpls.2026.1767742)

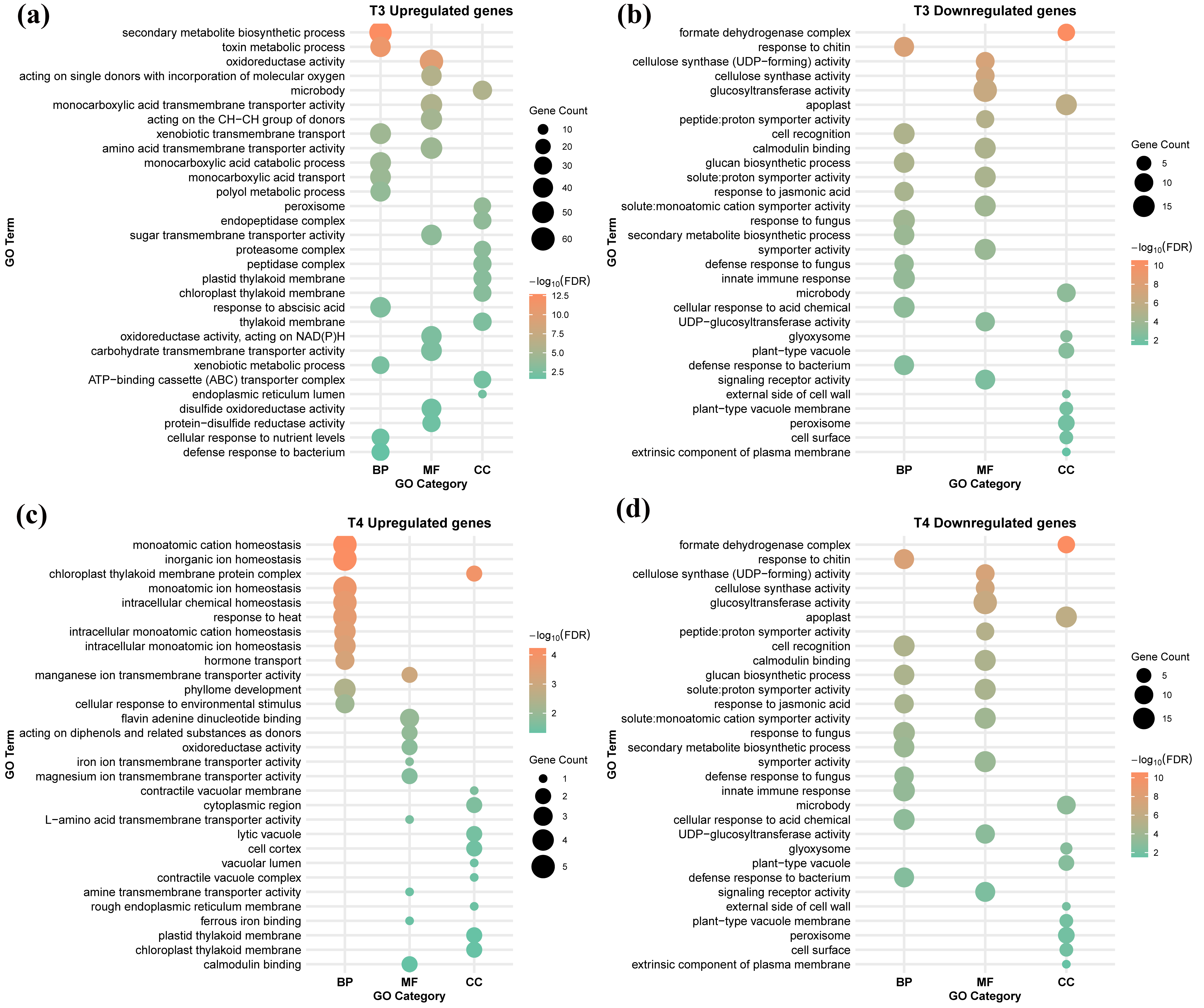

Supplement: Supplementary Figure 1 — Gene Ontology (GO) enrichment analysis of Differentially Expressed Genes (DEGs) for MT mitigation and the direct GSH vs. MT comparison. [file Image1.tif]

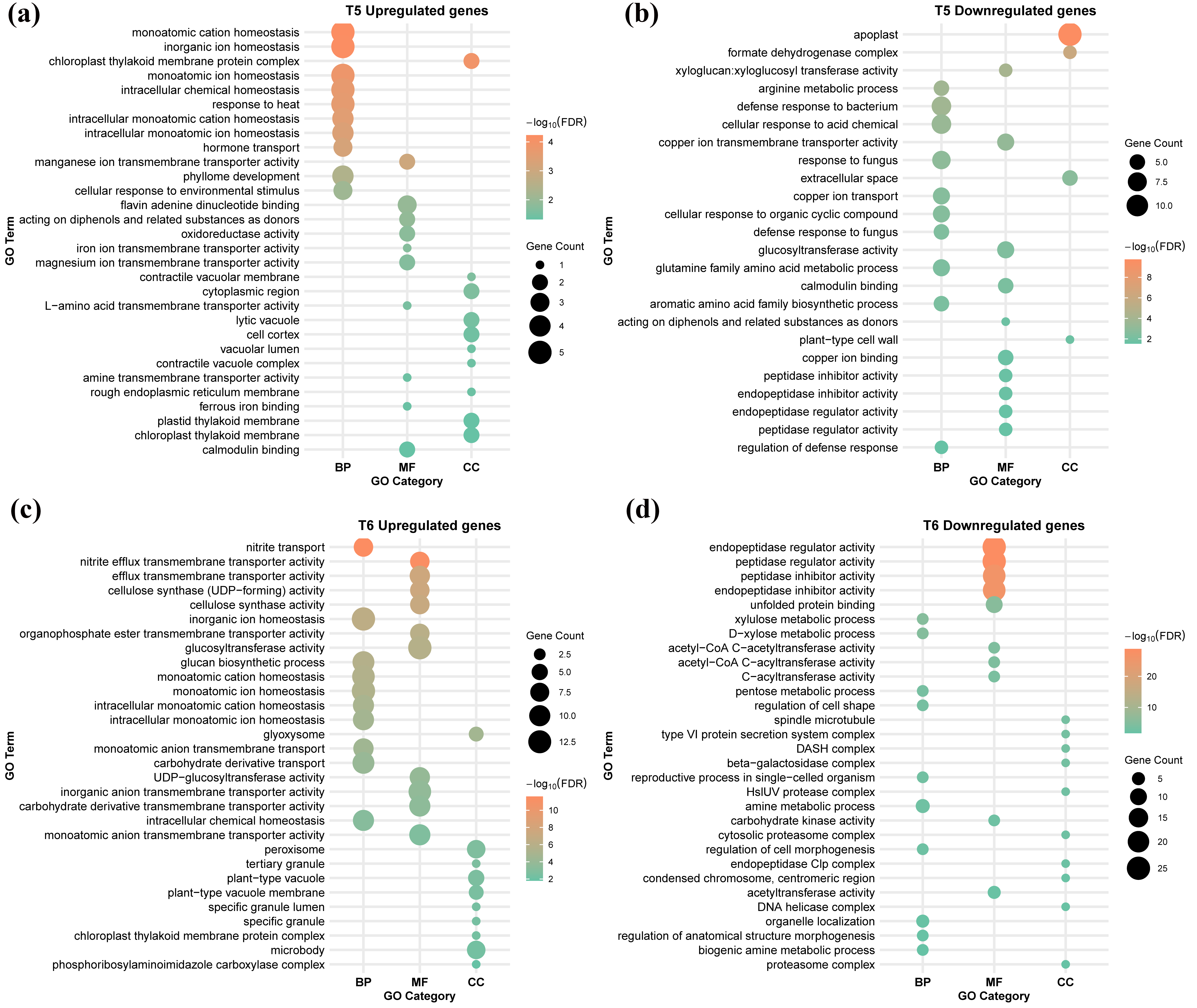

Supplement: Supplementary Figure 2 — Gene Ontology (GO) enrichment analysis of DEGs showing the overall effect of MT and GSH on the transcriptome relative to the CK. [file Image2.tif]

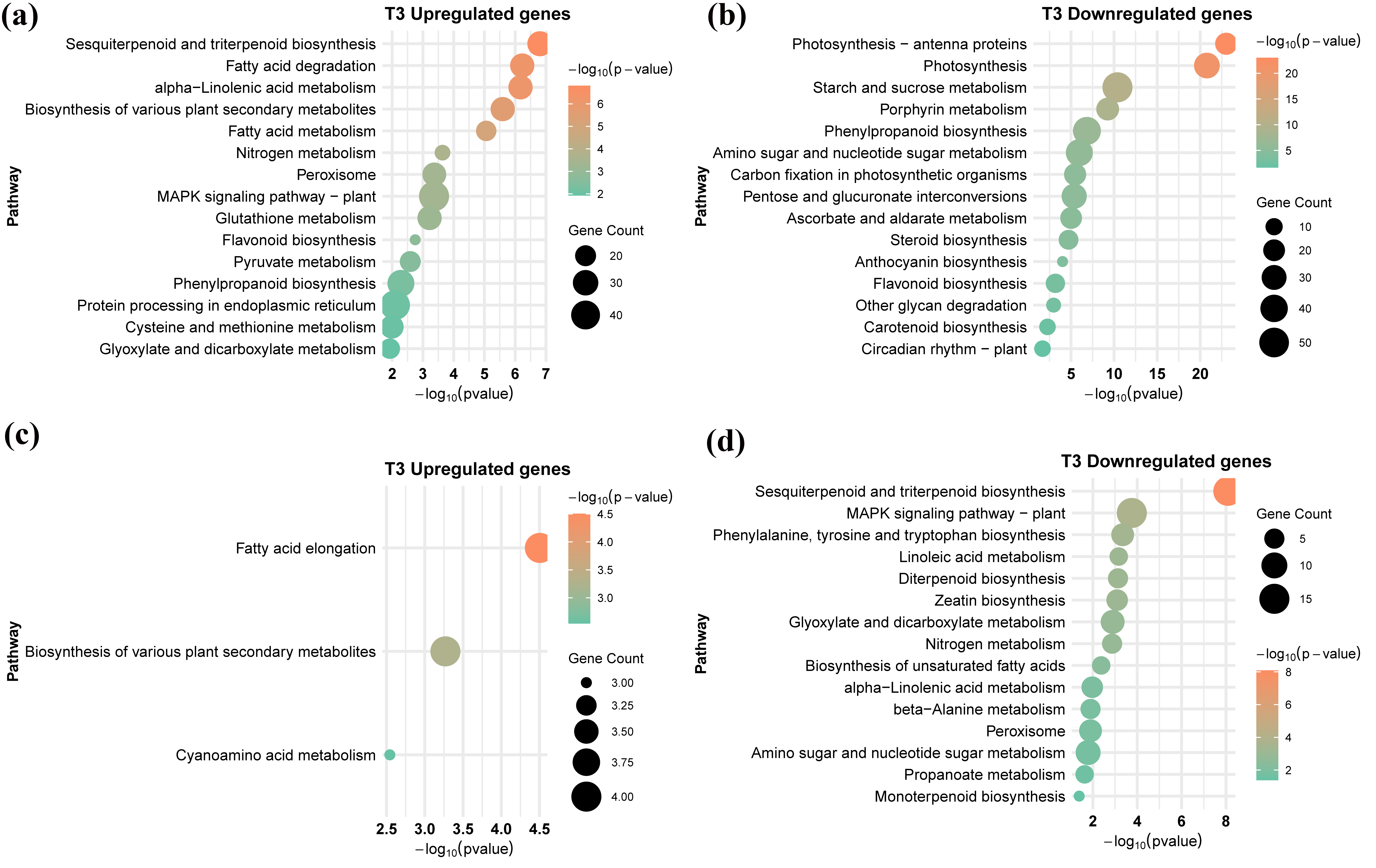

Supplement: Supplementary Figure 3 — KEGG pathway enrichment analysis of DEGs for MT mitigation and the direct GSH vs. MT comparison. [file Image3.tif]

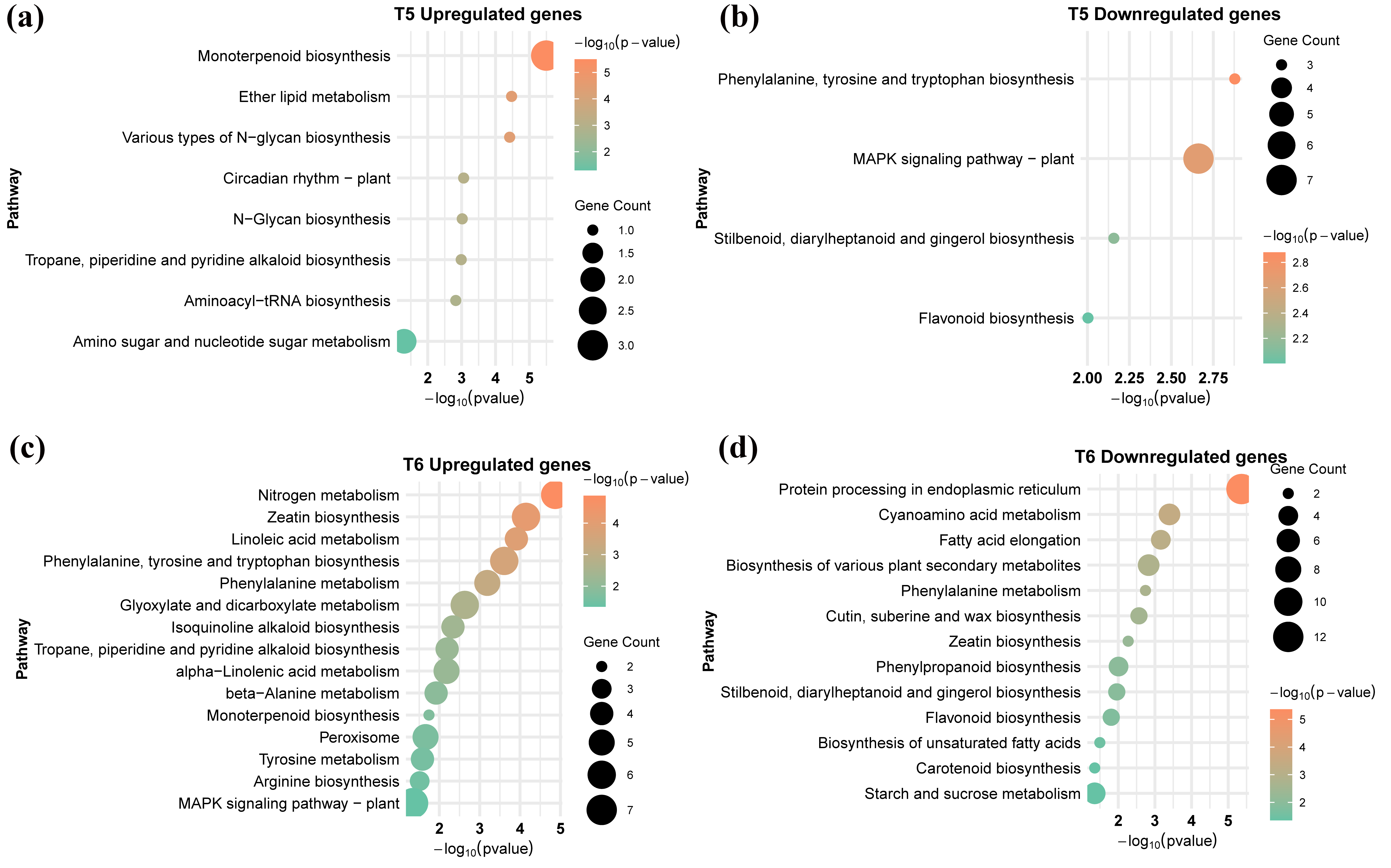

Supplement: Supplementary Figure 4 — KEGG pathway enrichment analysis of DEGs showing the overall effect of MT and GSH on the transcriptome relative to the CK. [file Image4.tif]
